# Supplementary material for: Using machine learning to simultaneously quantify multiple cognitive components of episodic memory
Source: Nat Commun. 2025 Mar 24;16:2856. doi: 10.1038/s41467-025-58265-9 (PMC11933255; doi:10.1038/s41467-025-58265-9)
Supplement: Supplementary file 1 — Supplementary Information [file 41467_2025_58265_MOESM1_ESM.pdf]

Supplementary Information for

Using machine learning to simultaneously quantify multiple cognitive components of episodic memory

Soroush Mirjalili, PhD., Audrey Duarte, Ph.D.

Corresponding author. Email: soroushmirjalili@utexas.edu

Supplementary Methods

Visual perception task

To behaviorally quantify each participant's perception (for excluding outlier participants) performance, the accuracy of the responses was used. The high/low labeling of the events for classification analyses is mentioned in the main text. For classification analyses, across subjects, 205 trials were associated with high perception (range of 106 to 255, SD = 33) and 64 trials were associated with low perception (range of 27 to 119, SD = 20).

Sustained attention task

To behaviorally quantify each participant's sustained attention performance (for excluding outlier participants), in addition to the response accuracy, the standard deviation of response time for correct responses divided by the mean response time and the accuracy of the responses were used. We divided the standard deviation response time by the mean response time to control for variability for participants' processing speed. The high/low labeling of the events for classification analyses is mentioned in the main text. For classification analyses, across subjects, 91 trials were associated with high sustained attention and 91 trials were associated with low sustained attention (range of 44 to 96, SD = 8 for both conditions).

Selective attention task

To behaviorally quantify each individual's selective attention performance (for excluding outlier participants), in addition to the response accuracy, the difference of standard deviation response time for the invalid and in the valid conditions will be divided by the mean response time during both conditions. We divided the difference by the mean response time to control for

variability for participants' processing speed and to highlight the impact of invalidity on performance. The high/low labeling of the events for classification analyses is mentioned in the main text. For classification analyses, across subjects, 114 trials were associated with high selective attention and 114 trials were associated with low selective attention (range of 84 to 119, SD = 6 for both conditions).

Episodic memory task

To behaviorally quantify each participant's item memory performance, item memory d' was used. To behaviorally quantify each participant's attended context memory performance (regardless of whether it was the color or the scene), attended context memory d' was computed as Z (proportion of "match" responses to contexts that matched those presented at encoding) – Z (proportion of match responses to contexts that mismatched those shown at encoding). Across subjects, there were 160 item hits (range of 76 to 216, SD = 30) and 56 item misses (range of 21 to 113, SD = 23), 104 context correct decisions (range of 59 to 177, SD = 30) and 55 context incorrect decisions (range of 16 to 116, SD = 30).

Control analyses for validating the transfer learning results

The first control analysis tested the importance of using meaningful sources predicted to support memory encoding. Our transfer learning approach assumed that each source would contribute to episodic encoding success such that episodic memory hits are more likely to have higher engagement from that cognitive source than are episodic memory misses. As a control for this hypothesis, we randomized the source labels and repeated the transfer learning procedure.

The second control analysis tested the importance of using the training portion of the encoding data to make the necessary adjustments when transferring a source to the target. As

previously mentioned, α and β are the regularized parameters that need to be calculated (i.e., during cross validation) to effectively transfer the source information to the target data by making the appropriate adjustment to the selected CSP-based features. Previously, we mentioned how choosing $\alpha = 0$ and $\beta = 0$ would result in the typical unidimensional approach for the classification of the target task which totally ignores the information from the source. Similarly, by choosing $\alpha = 1$ and $\beta = 0$, transfer learning completely ignores the target information and makes no adjustments to transfer the source information to the target data. And that's what we did in this control analysis to inspect the importance of using the training portion of the encoding data to make the appropriate adjustments to effectively transfer a source to the target.

Confirming dissociability of the 3 sources

We combined the events of all these three tasks and performed a 3-class classification. The label of each event was the task it belonged to (regardless of whether that event was a high or low performance event). We used a one versus rest voting approach to generalize the binary classification into a 3-class classification[1]. To elaborate, the first binary classifier would classify sustained attention events vs other events (perception and selective attention collapsed as a single class). Similarly, the second binary classifier would classify selective attention events vs other events, and the third binary classifier would classify perception events vs other events. The classifier evidence scores for these three binary classifiers would be combined to collectively predict which task an event belonged to. For each binary classification analyses performed here, we used the same procedure we used for other analyses which we described above (i.e., extracting CSP-based features from voltage and the power of different frequency bands, using a combination of the filter and wrapper methods to select the best 5 features among the 40 filtered features prior to training a naïve Bayes classifier).

Supplementary Notes

Behavioral Results

Behavioral performance associated with each task is shown in Supplementary Table 1.

Importance of different cognitive functions throughout the encoding period

We explored the extent to which different cognitive functions were involved during different encoding periods and how they predicted encoding success. We ran a source (perception, sustained attention, selective attention) × time window (early encoding [0-500 ms], middle encoding [500-1000ms], and late encoding [1000-1500 ms]) ANOVA for the associated regression weights (Supplementary Fig. 10B). The ANOVA results showed no significant main effects of cognitive function or time window and no significant interaction effect [all $Fs < 1.26$, all $ps > 0.287$, all $\eta_p^2 s < 0.001$]. These findings suggest that there is not a specific cognitive function during a specific encoding period which is consistently an outstanding factor in predicting memory success compared to other cognitive functions and encoding periods. To put it another way, on average across all participants, the difference between the levels of the 3 cognitive functions for hits and misses are stable across time and similar to each other as can be seen in Supplementary Fig. 10C.

Importance of different brain areas in the classification results

Even though EEG does not have high spatial resolution, it is vital to evaluate the importance of data recorded from different electrodes for determining memory success. As a brief reminder, this study extracted features based on CSP. Importantly, once a source is transferred to the target, the final set of CSP weights indicate how much the data recorded from each electrode was

important or relevant to transfer the source information to the target data. In each analysis, 5 CSP-based features for each participant were selected during each of 14 encoding time intervals and we took an average of the CSP weights across all selected features and participants. To get an insight of the importance of each brain area for each classification analysis, the associated average topography of the assigned weights to all brain areas is shown in Supplementary Fig. 9.

To determine the level of perception during encoding, the data recorded from the posterior areas received the highest weights. This is consistent with the idea that the dorsal and ventral visual systems are essential for perceiving a stimulus—regardless of the task nature—which is an essential step for successfully encoding an event[7,8]. Additionally, to predict the sustained attention level during encoding, the data recorded from frontal, central, parietal, and posterior regions, mainly on the right side, were most important. Along these lines, Cabeza and colleagues suggested the critical role of frontal and parietal cortices to maintain the attention analyze the stimulus on-line[8]. Furthermore, they suggested the general tendency of vigilance to engage right area of the brain more than left side right which is accordant to the previous lesion studies that suggest the right hemisphere is dominant during sustained attention mechanisms [9]. On top of that, Mangels and colleagues highlighted the importance of sustained interaction between frontal and posterior areas of the brain for elaborative processing of the events to successfully encode them[10]. Lastly, the frontal region of the brain received the highest weight to evaluate the selective attention level during encoding. This is consistent with the finding of [7] which showed the importance of frontal cortex when selecting the relevant target features. Similarly, Mangels and colleagues suggested the role of prefrontal cortex in deploying selective attention mechanisms to control which incoming information should be processed into consciousness[10]. In parallel, the findings of [8,11,12] suggested the importance of anterior cingulate cortex (whose

activity can be captured from frontal electrodes) for target assessment and selecting the relevant features among the competing presented features.

Importance of different frequency bands and voltage in the classification results

Among the top selected features across all participants, to transfer visual perception to episodic encoding, 16.0% were obtained from voltage, 18.7% from theta, 23.4% from alpha, 7.6% from beta, and 34.3% from gamma frequency bands. To transfer sustained attention to episodic encoding, 16.0% were obtained from voltage, 17.6% from theta, 23.1% from alpha, 7.8% from beta, and 35.5% from gamma frequency bands. To transfer selective attention to episodic encoding, 16.5% were obtained from voltage, 17.4% from theta, 22.6% from alpha, 7.6% from beta, and 35.9% from gamma frequency bands.

The classification results to predict attended context memory success

We found that transfer learning significantly enhanced our ability to predict attended context memory success across participants compared to the traditional approach/unidimensional approach (from 68.5% to 78.3%) [$t(42) = 11.220, p < 0.001, \mathrm{one-tailed}, d = 0.56$; Supplementary Fig. 12A]. In addition, we inspected how much memory classification performance would improve by adding each source in a stepwise manner rather than including all 3 sources simultaneously. We added the sources in all six possible orders and the patterns of results were similar and thus, we report the average findings. We found that there was a 5.2% performance improvement after the first source was added, regardless of order. There was a 2.9% performance improvement once the second source was added followed by a 1.7% performance improvement once the third source was added (Supplementary Fig. 12A).

Statistically, we found that adding each source significantly improved memory classification performance [$\text{all } ts > 2.785, \text{all } ps < 0.005, \text{one} - \text{tailed}, \text{all } ds > 0.10$]. Moreover, the extent to which classification performance increased by adding a source decreased with each step [step 2 improvement compared to step 1: $t(42) = 1.946, p = 0.029, \text{one} - \text{tailed}, d = 0.50$ and step 3 improvement compared to step 2: $t(42) = 1.210, p = 0.117, \text{one} - \text{tailed}, d = 0.31$].

Notably, the color/scene contextual cues at encoding are different, and the nature of the associated judgement is different as well. And in terms of the behavioral performance, attended context d' for attend-scene trials is significantly higher than attended context d' for attend-color trials [$t(42) = 3.609, p < 0.001, \text{one} - \text{tailed}, d = 0.638$]. Thus, we investigated whether the transfer learning results differ for the attend-color vs. attend-scene trials. Please note that we repeated the classification analyses only using attend-scene or only using attend-color trials which is different than simply breaking down the above context memory classification results into two categories of attend-color and attend-scene. We added the sources in all six possible orders and the patterns of results were quite similar and thus, we report the average findings (Supplementary Fig. 12B). The main noteworthy differences are the unidimensional classification performances and the extent of benefit the two conditions get from transfer learning. Specifically, the unidimensional context memory classification significantly outperforms for attend-scene trials compared to attend-color trials [$t(42) = 3.368, p < 0.001, \text{one} - \text{tailed}, d = 0.241$]. In addition, adding the three sources benefited the attend-color trials more than the attend-scene trials [$t(42) = 2.371, p = 0.011, \text{one} - \text{tailed}, d = 0.407$]. It is also worth mentioning that the multidimensional classification performance for context memory is lower when the attend-color and attend-scene conditions are collapsed (compared to

investigating those conditions separately), emphasizing the difference between the nature of these two conditions.

Investigating selective attention as a multidimensional process

In these analyses, we used selective attention as the target domain and used visual perception, sustained attention, and episodic encoding as the sources. We found that investigating selective attention as a multidimensional process using transfer learning significantly enhanced our ability to predict the selective attention level across participants compared to the traditional approach/unidimensional approach (from 66.8% to 76.4%) [$t(42) = 13.351, p < 0.001, \text{one} - \text{tailed}, d = 1.97$; Supplementary Fig. 13]. In addition, we inspected how much classifying the selective attention brain states would improve by adding each source in a stepwise manner rather than including all 3 sources simultaneously. We found that adding the first and second source significantly improved the classification performance, regardless of the order in which the sources were added [$\text{all } ts > 4.588, \text{all } ps < 0.001, \text{one} - \text{tailed}, \text{all } ds > 0.41$]. However, unlike the memory classification results, the order in which the sources were added mattered for how much the third source impacts the selective attention classification performance. Specifically, when added as the third source, while visual perception [$t(42) = 4.049, p < 0.001, \text{one} - \text{tailed}, d = 0.32$] and sustained attention [$t(42) = 4.329, p < 0.001, \text{one} - \text{tailed}, d = 0.37$] significantly improved the selective attention classification performance, adding episodic encoding as the third source did not lead to a significant improvement [$t(42) = 1.170, p = 0.124, \text{one} - \text{tailed}, d = 0.08$]. This suggests that episodic encoding cannot explain unique variance of selective attention-related neural activity while the information related to visual perception and sustained attention has already been leveraged.

Furthermore, we investigated whether there would be a diminishing return every step a new source is added. Again, the order in which the sources were added mattered. We first compared the improvements at the first and second steps. We found that when visual perception or sustained attention was added as the first source, the extent to which classification performance increased by adding a source significantly decreased [$\text{all } ts > 3.099, \text{all } ps < 0.002, \text{one} - \text{tailed}, \text{all } ds > 0.70$]. However, when episodic encoding was added as the first source, the extent to which classification performance increased by adding a source did not significantly decrease [$\text{all } ts < 1.673, \text{all } ps > 0.050, \text{one} - \text{tailed}, \text{all } ds < 0.40$]. We then compared the improvements at the second and third steps. We found that when visual perception or sustained attention was added as the second source, the extent to which classification performance increased by adding a source significantly decreased [$\text{all } ts > 3.800, \text{all } ps < 0.001, \text{one} - \text{tailed}, \text{all } ds > 0.88$]. However, when episodic encoding was added as the second source, the extent to which classification performance increased by adding a source did not significantly decrease [$\text{all } ts < 1.532, \text{all } ps > 0.065, \text{one} - \text{tailed}, \text{all } ds < 0.37$].

The time-on-task effect on each three sources

For each source, we investigated whether the level of engagement of the associated cognitive function fluctuated depending on how long the participant had been performing the source task (Supplementary Fig. 14). We computed the slope of evidence values against the number of trials presented across subjects. To be consistent with the analyses conducted for the encoding task, we broke down each of the 4 blocks for each source task into 4 mini-blocks to end up with 16 mini-blocks for each source task. For the perception task, as the time-on-task increased, the level of perception did not significantly change for both high and low trials

$[-0.353 < \text{all } \rho s < -0.179, \text{all } ps > 0.180]$. For the sustained attention task, as the time-on-task increased, the level of sustained attention significantly decreased for both high and low trials $[\text{all } \rho s < -0.829, \text{all } ps < 0.001]$. For the selective attention task, as the time-on-task increased, the level of selective attention significantly decreased for both high and low trials $[\text{all } \rho s < -0.948, \text{all } ps < 0.001]$.

The history effect on each three sources

For each source, we tested whether an event is more likely to involve higher levels of the associated cognitive function when it is preceded and followed by a history of higher levels of engagement from that cognitive function (Supplementary Fig. 15). As such, for the sustained attention task, we found that the neural evidence of high levels sustained attention was higher for events preceded $[F(1,168) = 70.40, p < 0.001, \eta_p^2 = 0.295]$ and followed $[F(1,168) = 32.86, p < 0.001, \eta_p^2 = 0.163]$ by a high sustained attention trial compared to events preceded by a low sustained attention trial. Such effects were not found for the perception and selective attention tasks $[\text{all } Fs < 0.94, \text{all } ps > 0.333, \text{ all } \eta_p^2 s < 0.006]$.

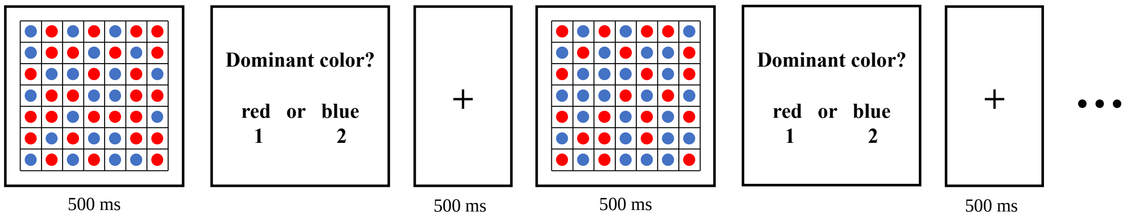

Supplementary Fig. 1. The perception task.

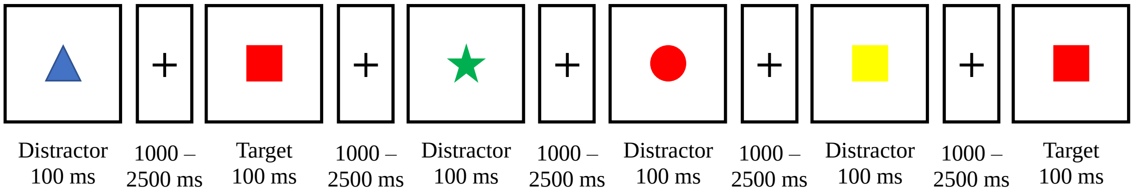

Supplementary Fig. 2. The Conjunctive Continuous Performance Test-Visual (CCPT-V) task. This task was used to assess the participants' level of sustained attention.

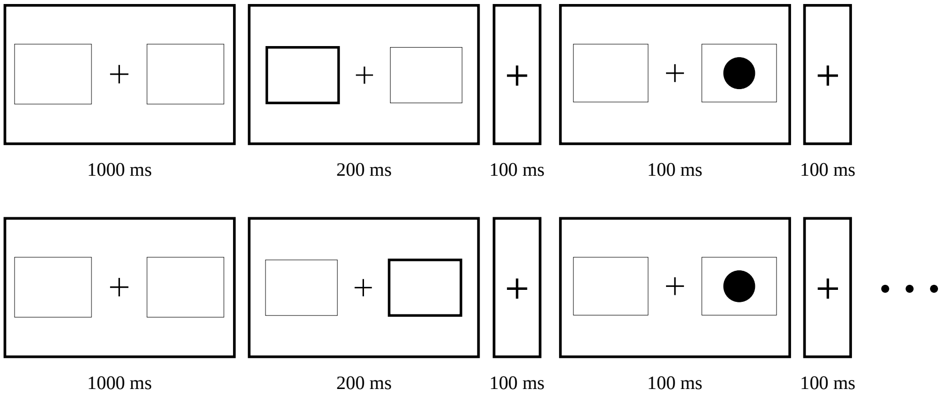

Supplementary Fig. 3. The Spatial Cued-Identification Task (SCIT) task. This task was used to assess the participants' level of selective attention.

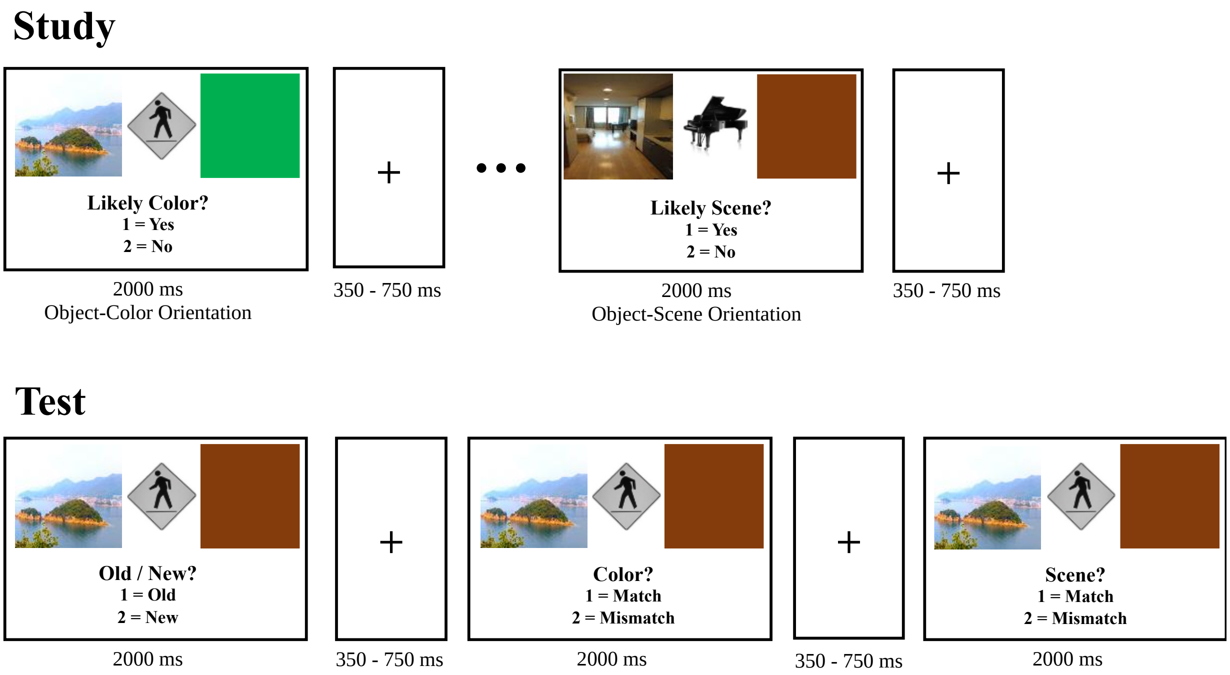

Supplementary Fig. 4. Task design for the episodic memory study. The scenes in this figure are taken from Creative Commons. The images are available under the following Creative Commons license: https://creativecommons.org/licenses/by/4.0/.

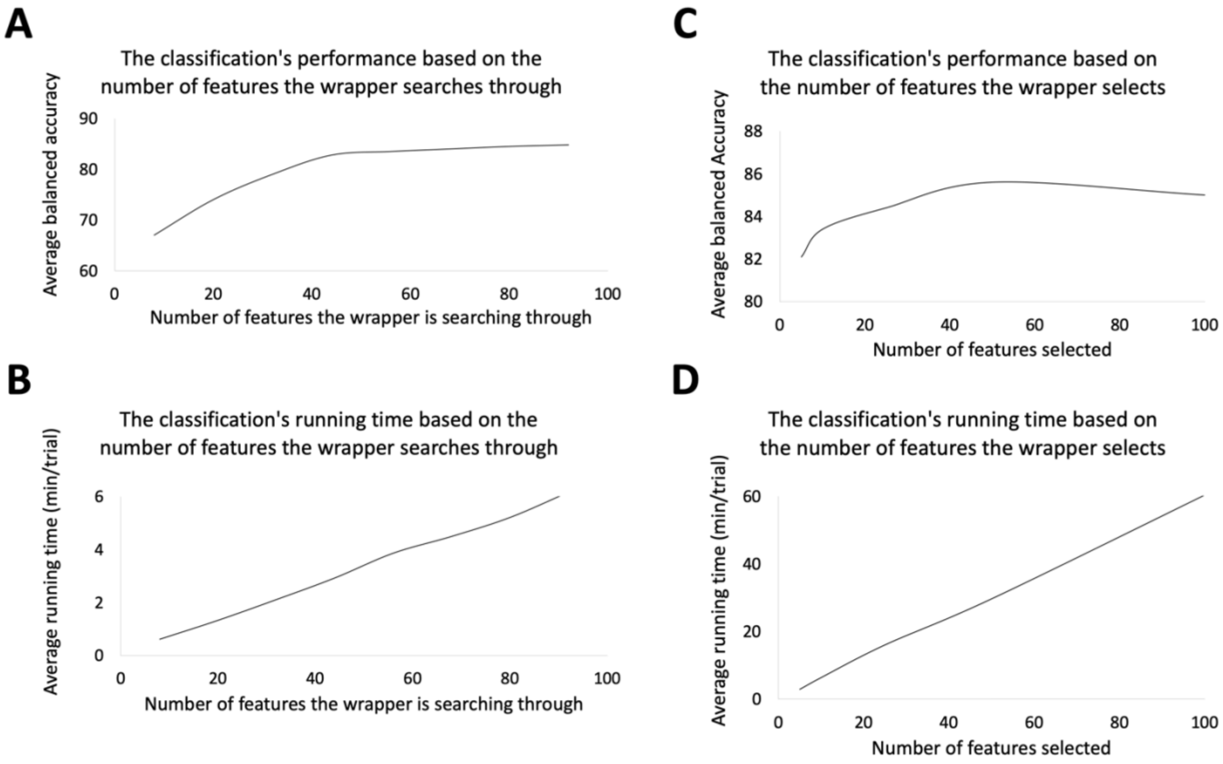

Supplementary Fig. 5. Determining the number of features to filter and select. The classification's **A)** performance and **B)** average running time based on the number of features the wrapper searches through (while selecting the top 5 features). The classification's **C)** performance and **D)** average running time based on the number of features the wrapper selects (among all 150 features). These results are obtained by using the data from 10 randomly selected participants. Source data are provided as a Source Data file.

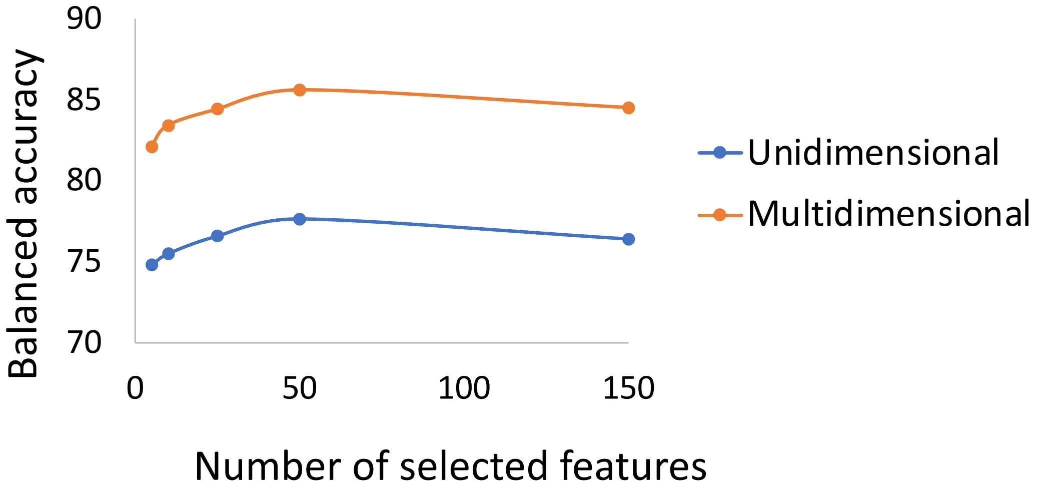

Supplementary Fig. 6. Classification results based on the number of selected features. Comparisons of balanced accuracy of the unidimensional vs. multidimensional approach for item memory brain states as a function of the number of selected features. Notably, the selected features were chosen among the 150 extracted features using the wrapper method. Source data are provided as a Source Data file.

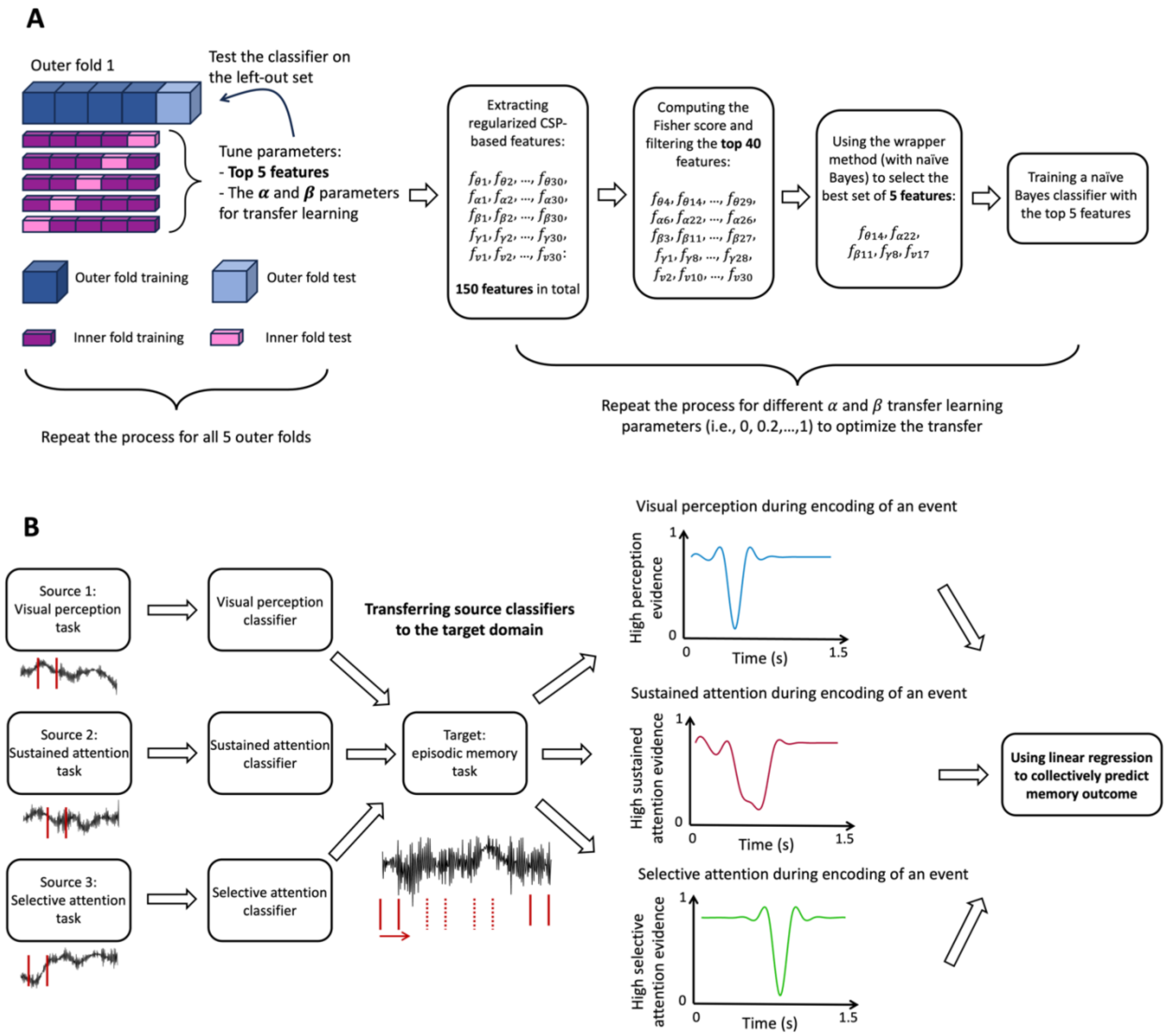

Supplementary Fig. 7. Description of the methodology. A) Here, we zoom into the details of the transfer learning procedure (i.e., transferring a source to a 200-ms encoding period). We used a nested cross-validation approach to ensure the left-out portion of the encoding data was never used during the training procedure. For each outer fold of the cross-validation, five-fold cross validation was performed for the training portion of the data to find the optimal parameters for classification. Using the voltage and the power time series over the 200-ms period (of both encoding and source data), we extracted 150 regularized CSP-based features. We then used the combination of the filter and the wrapper feature selection methods to reduce the number of features to 40 and then 5. After finding the top 5 features, we train a naïve Bayes classifier. **B)** An illustration of obtaining a temporal map of how high the brain state associated with each source is during an encoding event. The high vs. low performance classifier for each source was trained using an optimal 200 ms time window across participants. These optimal 200 ms time windows could be different across the three sources but for each source, the same 200 ms time window was used across all participants. Using a sliding time window approach, the information regarding the high vs low levels of each source was transferred to different encoding periods for

each event. This allowed us to determine the level of each source during different encoding periods for each event. Notably, the evidence for the low brain state for each source would be 1 - the evidence for the high brain state for that source. For example, if there is 0.8 evidence for high perception for the first 200 ms during an event's episodic encoding, there will be 0.2 evidence for low perception, but we have not showed that here to keep the figure as simple as possible. Once we obtain the temporal map corresponding to all 3 sources, we use linear regression to collectively predict memory outcome. We generated all the elements in this figure using PowerPoint and MATLAB.

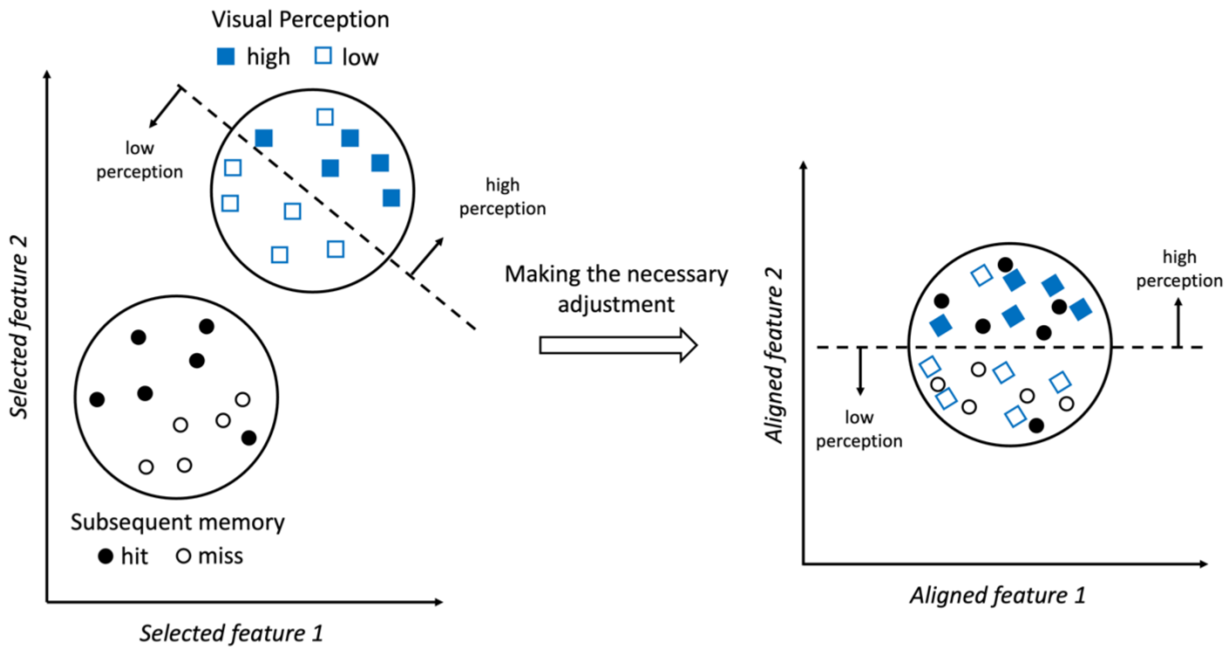

Supplementary Fig. 8. An illustration of how transfer learning makes the necessary adjustments to transfer a source to the target. The example in this figure is just for demonstrative purposes to provide an intuition of how transfer learning works. Specifically, when training a high vs. low perception performance classifier, we realize which CSP-based features (i.e., selected features 1 and 2 in this figure) can best distinguish high vs low perception events. The perception events are the blue squares and are shown in the selected 2-dimensional feature space. The decision boundary to separate high and low perception brain states is shown with a dashed line. Next, the same selected features will be extracted from the training portion of the encoding data and the encoding events will be projected into the selected feature space. However, when trying to determine the encoding events' perception level, the current decision boundary would label all the encoding events as low perception, suggesting the need to use transfer learning to make necessary adjustments. As such, transfer learning uses the information from the perception and the encoding data to project the data from both domains into a new aligned feature space that can effectively predict the perception level during encoding events.

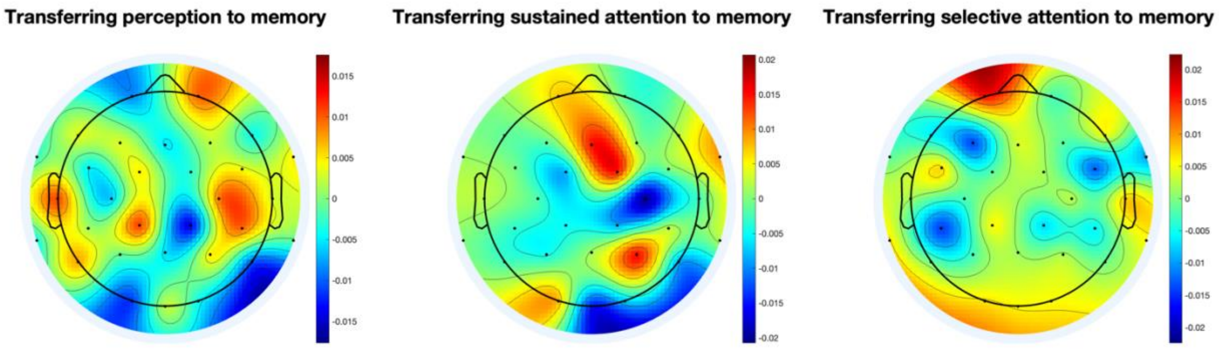

Supplementary Fig. 9. Getting an insight into the importance of different brain regions for different underlying processes during encoding. The topography of the weight the voltage and power associated with each electrode received when transferring each source to the encoding (on average across all participants). Source data are provided as a Source Data file.

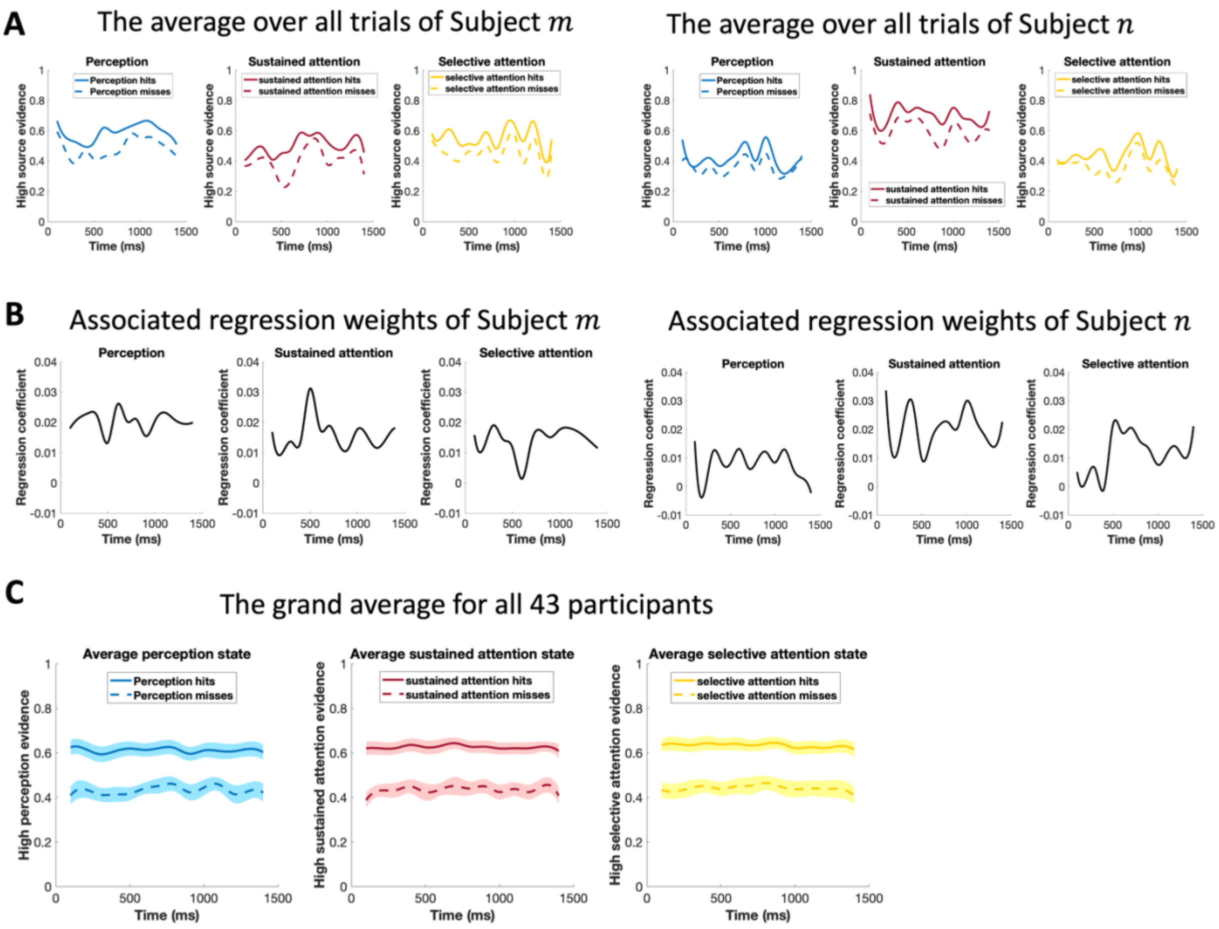

Supplementary Fig. 10. Getting an insight into the importance of different cognitive functions at different encoding periods for different individuals A) An example of subject-to-subject variation for the levels of perception, sustained, and selective attention during the encoding period for hits and misses. Subjects m and n are 2 arbitrary subjects chosen. **B)** The associated regression coefficients for each source at different encoding periods for subjects m and n. The output of the regression model is used to predict memory success. Notably, the regression coefficients provide important insight into which cognitive functions are particularly essential at different encoding periods for successful encoding for a participant. For example, the sustained attention processes during [400-600 ms] are particularly critical for successful encoding for subject m. **C)** The grand average of the levels of perception, sustained, and selective attention during the encoding period for hits and misses for all participants. The 95% confidence intervals are shown as well. Source data are provided as a Source Data file.

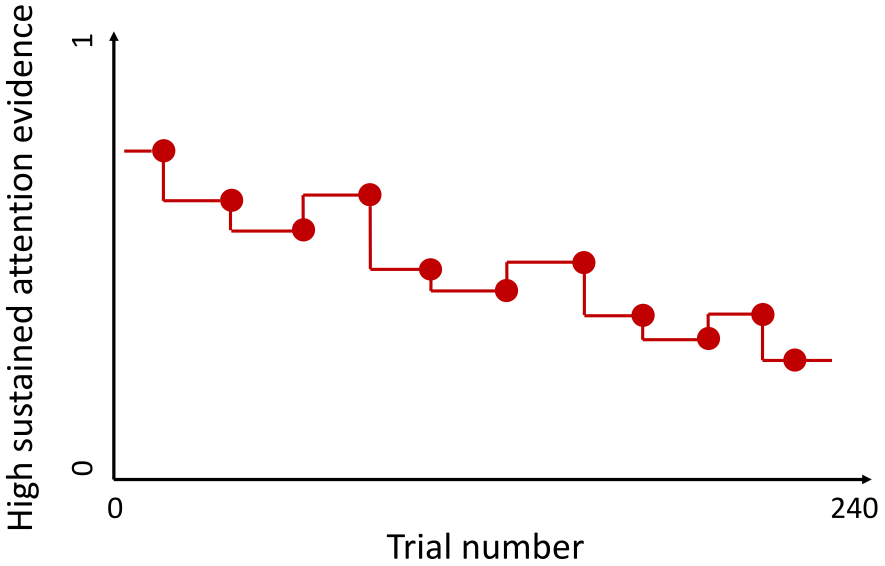

Supplementary Fig. 11. The interpolation approach to associate neural evidence to all 240 trials for each memory condition (i.e., hits and misses). In this figure, the red circles are the trials that are associated with the misses for that participant.

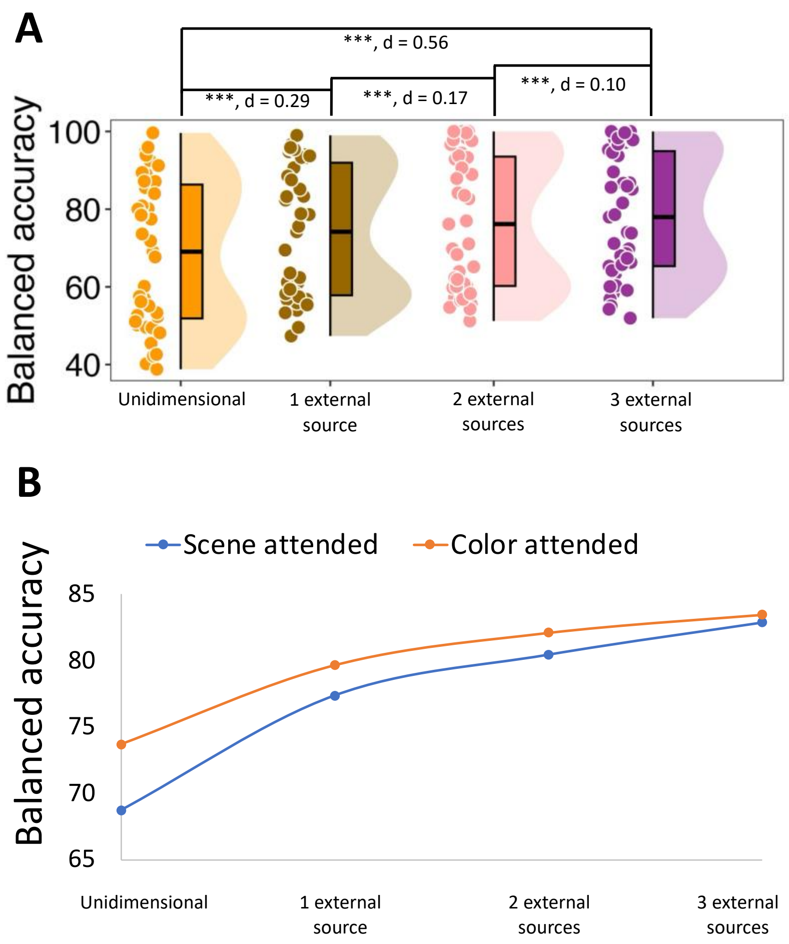

Supplementary Fig. 12. Attended context memory classification results. A) Comparisons of balanced accuracy for classifying attended context memory brain states as a function of how many of the sources are included (averaged across all six possible orders) during classification. Specifically, adding each source significantly improved the classification accuracy [$\text{all } ts > 2.785, \text{all } ps < 0.005, \text{one} - \text{tailed}, \text{all } ds > 0.10$]. Circles reflect the data points of individual participants (N = 43). In the box plots, the minima represent the lowest data point within a condition, maxima represent the highest data point, centre represent the median value within the box, bounds of the box are the 25th and 75th percentiles, whiskers extend from the box to the minimum and maximum values that are not considered outliers, and percentile refers to the position of a data point within the distribution, with the box representing the middle 50% of data points between the 25th and 75th percentiles. The asterisks reflect statistically significant differences using one-tailed tests across conditions using Holm-Bonferroni corrections for multiple comparisons (***p < 0.001) and the associated Cohen's d is shown for each comparison. **B**) The analyses were repeated only for trials in which the color was the attended context and separately for trials in which the scene was the attended context. To avoid complications and to make the comparisons simple, only the averages are shown. Source data are provided as a Source Data file.

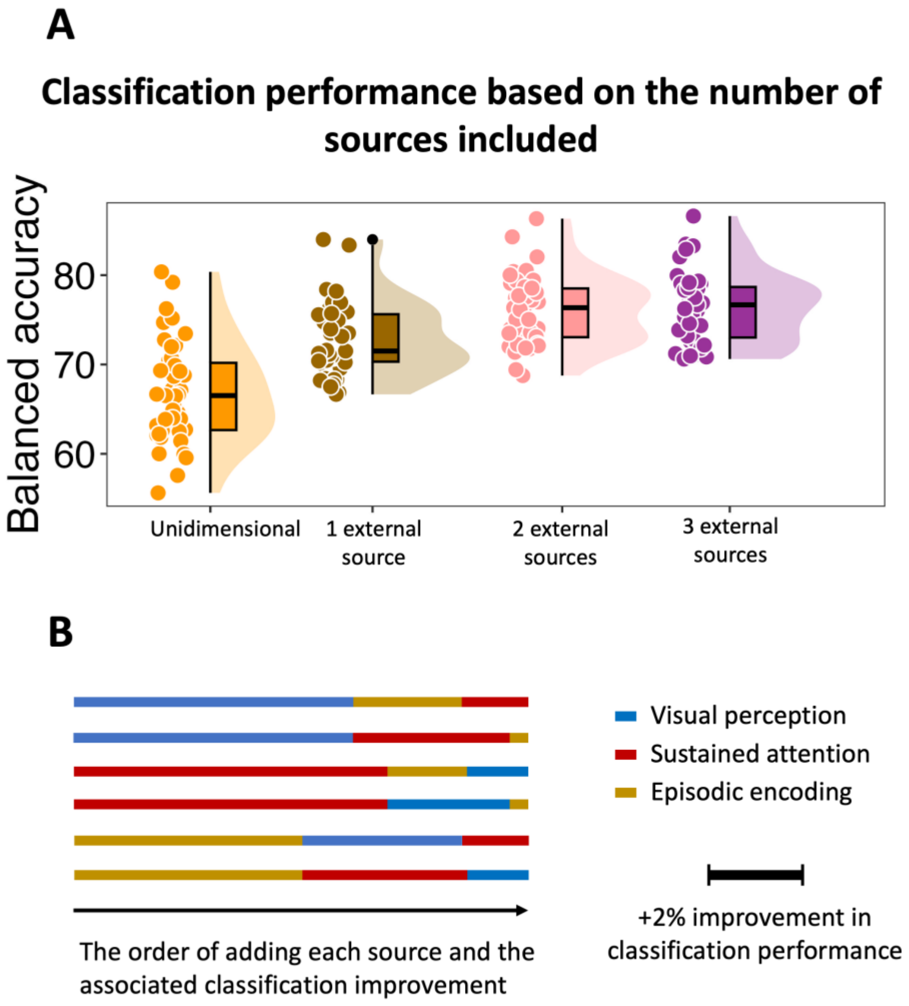

Supplementary Fig. 13. Investigating selective attention as a multidimensional process. (A) Comparisons of balanced accuracy for classifying selective attention brain states as a function of how many of the sources are included during classification. In the analysis associated with this figure, visual perception was added first, followed by sustained attention and then episodic encoding. Circles reflect the data points of individual participants (N = 43). In the box plots, the minima represent the lowest data point within a condition, maxima represent the highest data point, centre represent the median value within the box, bounds of the box are the 25th and 75th percentiles, whiskers extend from the box to the minimum and maximum values that are not considered outliers, and percentile refers to the position of a data point within the distribution, with the box representing the middle 50% of data points between the 25th and 75th percentiles. **(B)** The extent to which each added source improved the classification performance depending on the order in which the sources were added. Note that for memory classification results, we did not show this pattern as the results were very similar across the six possible orders the sources could be added. However, given the nature of the sources and the target in this particular analysis, the order in which the sources were added mattered and thus, the associated result for each possible order is shown. The length of each line represents the extent to which adding that source improved the classification performance. Source data are provided as a Source Data file.

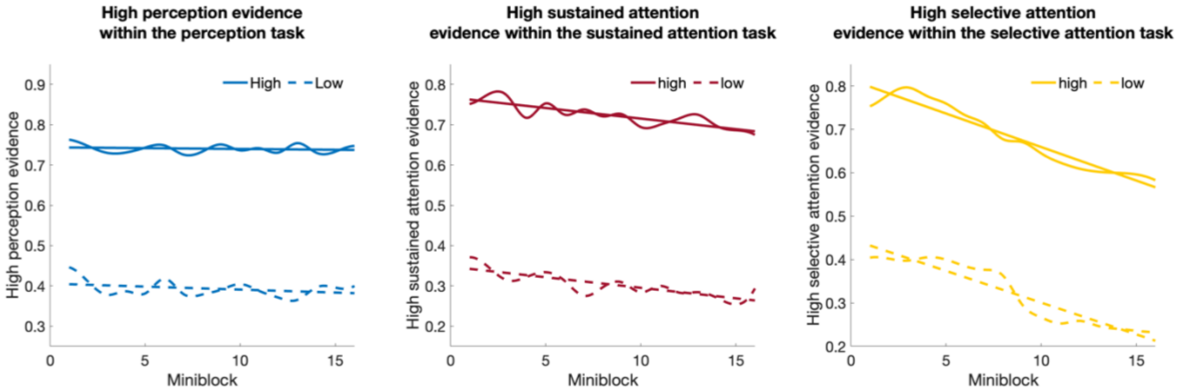

Supplementary Fig. 14. The time-on-task effect for the engagement of perception, sustained attention, and selective attention within each of their associated tasks. For each source, the neural evidence of high levels of that source for high and low performance trials within each mini-block was averaged for each participant. The average of these evidence scores for each source across all participants is shown separately for high and low performance trials. The associated lines of best fit are shown as well. Source data are provided as a Source Data file.

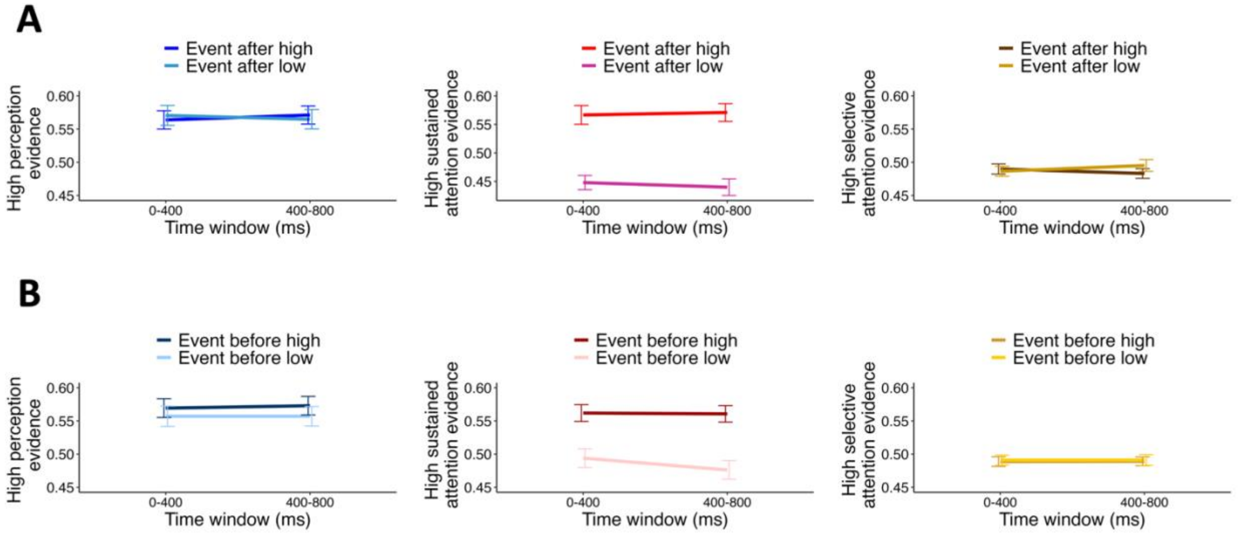

Supplementary Fig. 15. Impact of A) the prior and B) the subsequent event's associated brain state on the current event separately for each three sources. The current events are collapsed and averaged across high and low performance trials. The average level of engagement of the associated cognitive process for each source across all participants during early (i.e., [0-400 ms]), and late (i.e., [400-800 ms]) trial periods is shown. We conducted Memory condition × Source × Time ANOVA for the associated analyses. The main effect of Memory Condition was significant for sustained attention (prior event's effect: $[F(1,168) = 70.40, p < 0.001, \eta_p^2 = 0.295]$, subsequent event's effect: $[F(1,168) = 32.86, p < 0.001, \eta_p^2 = 0.163]$) but not for visual perception or selective attention [all $Fs < 0.94$, all $ps > 0.333$, all $\eta_p^2 s < 0.006$]. The associated error bars for each condition reflect standard error of the mean obtained across all participants (N = 43). Unlike the encoding task, and due to the shorter period for each stimulus for the source tasks, we had to consider only the first 800 ms of the trial's epoch for these analyses. Source data are provided as a Source Data file.

Item d'	$2.08 \pm 0.063\ (SE)$
Attended context d'	$1.65 \pm 0.064\ (SE)$
Perception	$74.64\% \pm 1.27\%\ (SE)$
Sustained attention mean response time	$451.0\ ms \pm 5.2\ ms\ (SE)$
Sustained attention performance	$92.29\% \pm 0.93\%\ (SE)$
Selective attention mean response time valid	$338.6\ ms \pm 6.9\ ms\ (SE)$
Selective attention mean response time invalid	$386.9\ ms \pm 6.5\ ms\ (SE)$
Selective attention performance	$90.70\% \pm 1.28\%\ (SE)$

Supplementary Table 1. The behavioral performance on the tasks associated with the sources and the target. SE stands for standard error. Source data are provided as a Source Data file.

Supplementary references

1. Mirjalili, S., Powell, P., Strunk, J., James, T. & Duarte, A. Evaluation of classification approaches for distinguishing brain states predictive of episodic memory performance from electroencephalography: Abbreviated Title: Evaluating methods of classifying memory states from EEG. *Neuroimage* **247**, (2022).
2. Zhu, J.-Y., Zheng, W.-L. & Lu, B.-L. Cross-subject and Cross-gender Emotion Classification from EEG. in *World Congress on Medical Physics and Biomedical Engineering, June 7-12, 2015, Toronto, Canada* (ed. Jaffray, D. A.) 1188–1191 (Springer International Publishing, Cham, 2015).
3. Prema, P., Kesavamurthy, T. & Arulmozhivarman, P. Event detection in single trial EEG during attention and memory related task. *Int J Biomed Eng Technol* **36**, 358–374 (2021).
4. Bernstein, L. J., Beig, S., Siegenthaler, A. L. & Grady, C. L. The effect of encoding strategy on the neural correlates of memory for faces. *Neuropsychologia* **40**, 86–98 (2002).
5. Berger, G. H. & Gaunitz, S. C. B. Self-rated imagery and encoding strategies in visual memory. *British Journal of Psychology* **70**, 21–24 (1979).
6. Leshikar, E. D., Duarte, A. & Hertzog, C. Task-Selective Memory Effects for Successfully Implemented Encoding Strategies. *PLoS One* **7**, e38160- (2012).
7. Machinskaya, R. I., Krupskaya, E. V & Kurgansky, A. V. Functional brain organization of global and local visual perception: Analysis of event-related potentials. *Hum Physiol* **36**, 518–534 (2010).
8. Cabeza, R. & Nyberg, L. Imaging Cognition: An Empirical Review of PET Studies with Normal Subjects. *J Cogn Neurosci* **9**, 1–26 (1997).
9. Pardo, J. V, Fox, P. T. & Raichle, M. E. Localization of a human system for sustained attention by positron emission tomography. *Nature* **349**, 61–64 (1991).
10. Mangels, J. A., Picton, T. W. & Craik, F. I. M. Attention and successful episodic encoding: an event-related potential study. *Cognitive Brain Research* **11**, 77–95 (2001).
11. Devinsky, O., Morrell, M. J. & Vogt, B. A. Contributions of anterior cingulate cortex to behaviour. *Brain* **118**, 279–306 (1995).
12. Pardo, J. V, Pardo, P. J., Janer, K. W. & Raichle, M. E. The anterior cingulate cortex mediates processing selection in the Stroop attentional conflict paradigm. *Proceedings of the National Academy of Sciences* **87**, 256–259 (1990).
