# Supplementary material for: Using machine learning to simultaneously quantify multiple cognitive components of episodic memory
Source: Nat Commun. 2025 Mar 24;16:2856. doi: 10.1038/s41467-025-58265-9 (PMC11933255; doi:10.1038/s41467-025-58265-9)
Supplement: Supplementary file 2 — Reporting Summary [file 41467_2025_58265_MOESM2_ESM.pdf]

nature portfolio

Corresponding author(s): Soroush Mirjalili

Last updated by author(s): 01/28/2025

Reporting Summary

Nature Portfolio wishes to improve the reproducibility of the work that we publish. This form provides structure for consistency and transparency in reporting. For further information on Nature Portfolio policies, see our Editorial Policies and the Editorial Policy Checklist.

Statistics

For all statistical analyses, confirm that the following items are present in the figure legend, table legend, main text, or Methods section.

n/a	Confirmed	
☐	☒	The exact sample size (*n*) for each experimental group/condition, given as a discrete number and unit of measurement
☐	☒	A statement on whether measurements were taken from distinct samples or whether the same sample was measured repeatedly
☐	☒	The statistical test(s) used AND whether they are one- or two-sided<br>*Only common tests should be described solely by name; describe more complex techniques in the Methods section.*
☒	☐	A description of all covariates tested
☐	☒	A description of any assumptions or corrections, such as tests of normality and adjustment for multiple comparisons
☐	☒	A full description of the statistical parameters including central tendency (e.g. means) or other basic estimates (e.g. regression coefficient) AND variation (e.g. standard deviation) or associated estimates of uncertainty (e.g. confidence intervals)
☐	☒	For null hypothesis testing, the test statistic (e.g. *F*, *t*, *r*) with confidence intervals, effect sizes, degrees of freedom and *P* value noted<br>*Give P values as exact values whenever suitable.*
☒	☐	For Bayesian analysis, information on the choice of priors and Markov chain Monte Carlo settings
☐	☒	For hierarchical and complex designs, identification of the appropriate level for tests and full reporting of outcomes
☐	☒	Estimates of effect sizes (e.g. Cohen's *d*, Pearson's *r*), indicating how they were calculated

Our web collection on statistics for biologists contains articles on many of the points above.

Software and code

Policy information about availability of computer code

Data collection	EEG using Brain Vision ActiCAP system
Data analysis	All the analyses were done in Matlab 2021b. R was used for making some of the manuscript's figures while Matlab and Excel were used for the remaining figures.

For manuscripts utilizing custom algorithms or software that are central to the research but not yet described in published literature, software must be made available to editors and reviewers. We strongly encourage code deposition in a community repository (e.g. GitHub). See the Nature Portfolio guidelines for submitting code & software for further information.

Data

Policy information about availability of data

All manuscripts must include a data availability statement. This statement should provide the following information, where applicable:

- Accession codes, unique identifiers, or web links for publicly available datasets
- A description of any restrictions on data availability
- For clinical datasets or third party data, please ensure that the statement adheres to our policy

All data and materials used in the analysis are available on https://osf.io/8vfy4/.

Research involving human participants, their data, or biological material

Policy information about studies with human participants or human data. See also policy information about sex, gender (identity/presentation), and sexual orientation and race, ethnicity and racism.

Reporting on sex and gender	The participants consisted of 47, right-handed adults (22 men, 24 women, 1 non-binary). The non-binary person was biologically born as a female. Moreover, sex and/or gender was not considered in the study design and sex and/or gender of participants was determined based on self-report. We did not perform any sex or gender-related analyses as there is no hypothesis suggesting differences based on sex or gender related to this study.
Reporting on race, ethnicity, or other socially relevant groupings	The sample consisted of a racially diverse group of people. There were 11 White, 2 Black or African American, 1 American Indian or Alaska Native, 19 Asian, 9 Hispanic or Latino, and 5 Multiracial participants.
Population characteristics	The participants consisted of 47, right-handed adults from ages 18 to 35. All participants were fluent in English and had normal or corrected vision. Except for one participant diagnosed with PTSD (that we could not find out about during initial screening), none of the other participants reported any psychiatric or neurological disorders, vascular disease, or use of any type of medication that affects the central nervous system.
Recruitment	All participants were recruited from the University of Texas at Austin and surrounding community. Fliers were the main way to recruit participants. There was no self-selection bias that could impact the results.
Ethics oversight	University of Texas at Austin Institutional Review Board.

Note that full information on the approval of the study protocol must also be provided in the manuscript.

Field-specific reporting

Please select the one below that is the best fit for your research. If you are not sure, read the appropriate sections before making your selection.

☒ Life sciences ☐ Behavioural & social sciences ☐ Ecological, evolutionary & environmental sciences

For a reference copy of the document with all sections, see nature.com/documents/nr-reporting-summary-flat.pdf

Life sciences study design

All studies must disclose on these points even when the disclosure is negative.

Sample size	Given the novelty of this study, we could not predict an effect size for our analyses. The sample size was selected based on the other method comparison EEG studies in the cognitive neuroscience field.
Data exclusions	We excluded 4 participants: 3 of them were outliers in terms of performance in at least 2 of the tasks and the other person had post-traumatic stress disorder (PTSD) even though we had not realized that during initial screening.
Replication	The code and data are provided in the following link which makes this study to be easily replicable: https://osf.io/8vfy4/
Randomization	Participants were not allocated into between-subject experimental groups.
Blinding	Not applicable, there was only one group of interest and there was no manipulation performed (e.g., it was not the case that the perception task looked different for half of the participants)

Reporting for specific materials, systems and methods

We require information from authors about some types of materials, experimental systems and methods used in many studies. Here, indicate whether each material, system or method listed is relevant to your study. If you are not sure if a list item applies to your research, read the appropriate section before selecting a response.

Materials & experimental systems

n/a	Involved in the study
☒	☐ Antibodies
☒	☐ Eukaryotic cell lines
☒	☐ Palaeontology and archaeology
☒	☐ Animals and other organisms
☒	☐ Clinical data
☒	☐ Dual use research of concern
☒	☐ Plants

Methods

n/a	Involved in the study
☒	☐ ChIP-seq
☒	☐ Flow cytometry
☒	☐ MRI-based neuroimaging

Plants

Seed stocks	*Report on the source of all seed stocks or other plant material used. If applicable, state the seed stock centre and catalogue number. If plant specimens were collected from the field, describe the collection location, date and sampling procedures.*
Novel plant genotypes	*Describe the methods by which all novel plant genotypes were produced. This includes those generated by transgenic approaches, gene editing, chemical/radiation-based mutagenesis and hybridization. For transgenic lines, describe the transformation method, the number of independent lines analyzed and the generation upon which experiments were performed. For gene-edited lines, describe the editor used, the endogenous sequence targeted for editing, the targeting guide RNA sequence (if applicable) and how the editor was applied.*
Authentication	*Describe any authentication procedures for each seed stock used or novel genotype generated. Describe any experiments used to assess the effect of a mutation and, where applicable, how potential secondary effects (e.g. second site T-DNA insertions, mosiacism, off-target gene editing) were examined.*
